# Sensorless control of dual three-phase permanent magnet synchronous motor based on speed feedback and frequency-variable tracking

**DOI:** 10.1371/journal.pone.0294728

**Published:** 2023-11-28

**Authors:** Hanying Gao, Yao Dong

**Affiliations:** School of Electrical and Electronic Engineering, Harbin University of Science and Technology, Harbin, China; University of Bradford, UNITED KINGDOM

## Abstract

The dual three-phase Permanent Magnet Synchronous Motor (PMSM) control system is characterized by its high reliability, slight torque fluctuation and low harmonic content. It is very suitable for systems requiring high power output and high reliability, for instance, electric vehicles, aerospace and military equipment. In this paper, a full speed domain sensorless control technology for dual three-phase PMSM is proposed which solves the limitations of other sensorless controls, improves system accuracy and stability, and has high practicality in fields such as new energy vehicles. The mathematical model of this motor in a static coordinate system is established, and the sine and cosine signals along with the velocity and angle information are obtained by using the flux linkage observer. Moreover, the estimated angle error parameter is introduced into the flux linkage observer; as a result, the estimation accuracy is improved by the estimated speed feedback, and the current frequency is tracked by the stator current Frequency-Variable Tracker (FVT) to reduce the current error. Meanwhile, to make the observer’s estimation more accurate and to improve its ability to resist disturbance, a rotor disturbance is added to act as a disturbance variable. Through the mechanical motion equation of the motor, a fourth-order Extended State Observer (ESO) is built to calculate the rotor position and speed. Finally, the technology accuracy is verified using simulation and experimental results. The findings prove that the sensorless detection technology, with speed feedback introduce in this paper, has good reliability and high precision for dual three-phase PMSM under dynamic and static conditions.

## 1 Introduction

Multi-phase Permanent Magnet Synchronous Motor (PMSM) is characterized by its low torque ripple, high efficiency, low voltage, high-power speed regulation and strong fault tolerance. The multi-phase PMSM-based drive system is very useful in land traffic, ships, aerospace and other application fields [1–3]. The multi-phase PMSM vector control system usually needs to integrate the position sensor to detect the rotor position and the speed information. However, the use of position sensors increases the cost and size, while also reducing the reliability and safety of the system. Consequently, to improve reliability, the sensorless detection technology is a feasible technical solution, which has essential research significance [4–6]. Moreover, the multi-phase PMSM sensorless detection technology is generally divided into two categories: high-frequency injection method and mathematical model method. The first method is to estimate the rotor speed and its position by applying the salient pole effect of the rotor; which is suitable for low and zero speed states [7, 8]. Its calculation precision is irrelevant to the motor speed and is insensitive to parameter changes; however, it has certain saliency requirements for salient polarity of permanent magnet synchronous motors. Moreover, if the high-frequency interference signal is not set properly, the system will produce an electromagnetic noise. At the same time, its operating range and dynamic characteristics are also limited by the bus voltage. The second category is applicable to motors in medium to high speed regions, where the estimation of the speed and rotor position depends on the motor back Electro Magnetic Force (EMF), such as the sliding mode observer method [9], the model reference method [10] and the sliding mode observer method [11, 12].

In recent years, sensorless control have received widespread attention and have received extensive research on accuracy issues. The author of [13] proposed a fractional-order Phase-Locked Loop (PLL) is proposed to replace the traditional PLL in the sliding mode observer, the rotor position can be accurately estimated by reasonably selecting the fractional order coefficient. However, when the motor parameters change due to changes in operating state, there is a lack of analysis of system robustness. In [14], the soft phase-locked loop is used to eliminate the deviation caused by the low-pass filters and the phase shift compensation link in the sliding mode observer for dual three-phase PMSM, so as to improve the detection accuracy of the rotor position and speed. The study presented in [15] proposed an integral sliding mode observer to compensate the estimated back EMF error. Despite these two types of observers eliminate the problems of vibration and the phase delay, it has poor applicability in case of load disturbance and cannot be widely used. In contrast, the high-frequency injection method has higher practicality and practical significance. The study presented in [16] not only the method of injecting high-frequency square wave voltage is used, but also a new extended state observer is applied to observe the motor speed and its position. Compared to the traditional method, this observer reduces the use of digital filters, and it reduces the information processing steps and eliminates the error caused by the nonlinearity of the inverter. However, the position estimation accuracy obtained by this method is limited and it is only applied to motors running at low speeds. The author of [17] propose an application of zero-sequence carrier voltage improved pulse signal injection method in dual three-phase PMSM, leading to improve the accuracy of position detection. That is, to inject two high-frequency signals into two sets of windings independently, and through the optimal angle between the signals, an adjusted rotating carrier signal injection method based on zero-sequence voltage without a position sensor, is proposed, can effectively suppress position estimation errors caused by sixth harmonic generation. Reference [18] proposed a rotor position estimation method based on back EMF, which alleviated the influence of the magnetic saturation and temperature variations on the estimated rotor flux position by directly measuring the back EMF. Based on the principles of induced electromotive force and harmonic current in dual three-phase PMSM, reference [19] analyzed the impact of sensorless detection technology based on stator flux observer and simplified extended Kalman filter on observation accuracy, and conducted indepth research. The experimental results show that the simplified extended Kalman filter position sensorless detection technology exhibits better accuracy in position and velocity estimation; in terms of dynamic performance, compared with the stator flux observer based sensorless control technology, the estimation results of the simplified extended Kalman filter position free sensor detection technology have less phase lag and fluctuation phenomenons. In [20], the switching table of the direct torque control is optimized to reduce the harmonic current of the stator; further more, it adopts a simplified extended Kalman filter for improving the position-free observation accuracy of the dual three-phase PMSM. However, the above methods did not solve the inapplicability of the mathematical model of the motor at low speeds.

In this paper, a dual three-phase PMSM with two sets of windings separated by a neutral point with a difference of 30° electrical angle is studied. Thus, a new sensorless detection technique with speed feedback and a rotor position estimation method is adopted, it can achieve high-precision sensorless control of the motor in the full speed domain, addressing the limitations of high-frequency injection method and mathematical model method, and has good dynamic performance. First, the sine and cosine signals with velocity and angle information are obtained by using the flux linkage observer; this latter, removes the influence of the flux linkage and rotational speed com-pared with the back EMF. Moreover, the estimated angle error parameter is introduced into the flux linkage observer where the estimation accuracy is improved by the estimated speed feedback, and the current frequency is tracked using the stator current FVT to reduce the current error. Simultaneously, a fourth-order Extended State Observer (ESO) is built through the mechanical motion equation of the motor, and for the sake of ensuring its anti-interference ability and precision, the rotor position angle error consists of a whole disturbance variable. Finally, the simulation model of the observer system is built using software techniques and the hardware experimental platform is deployed to verify the accuracy and effectiveness of this observer. Through the experimental and simulation results, it is concluded that the sensorless control system with speed feedback has strong disturbance resistance and fast estimation speed.

## 2 Dual-phase PMSM motor model

**Fig 1:**
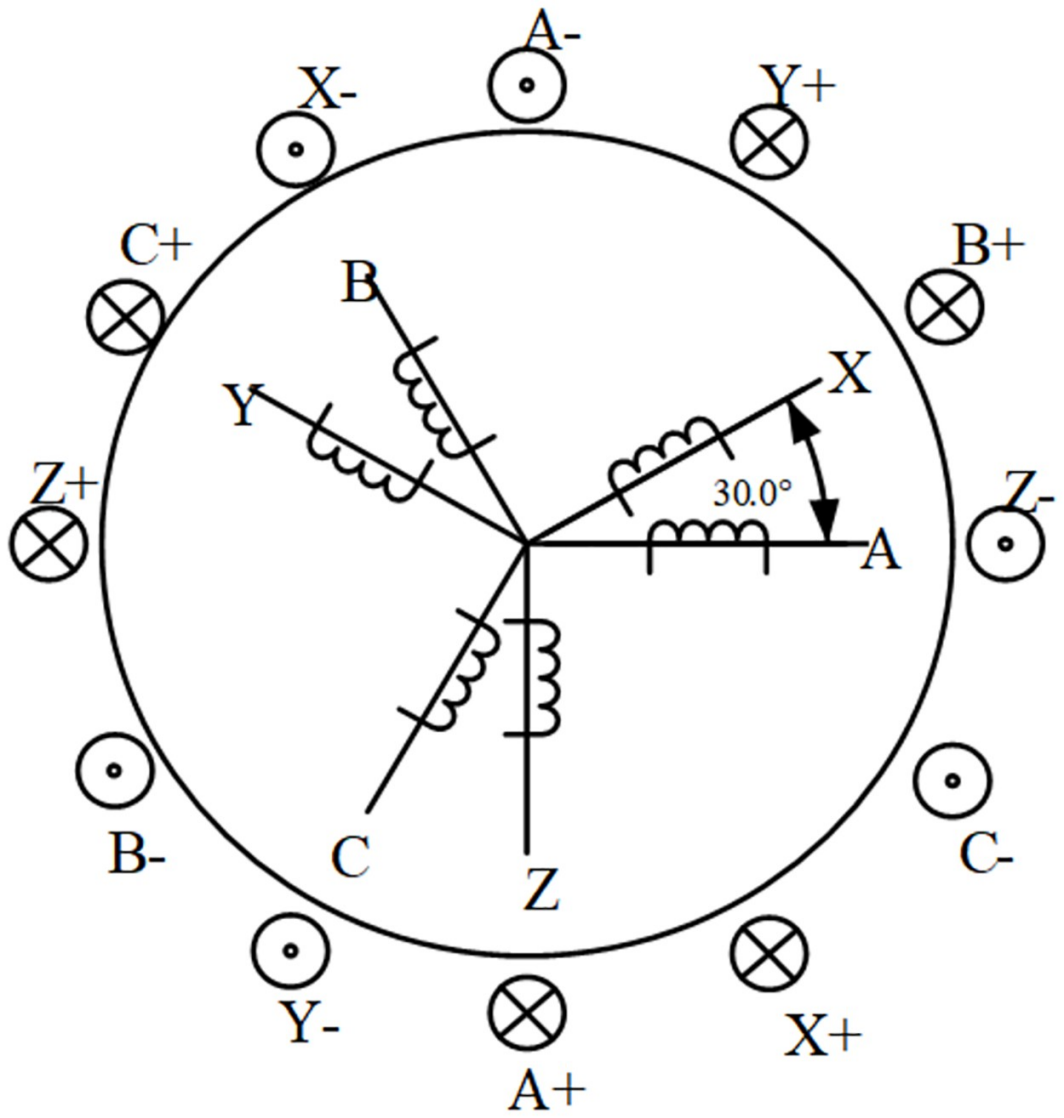
Distribution diagram of dual three-phase PMSM windings.

The distribution of dual three-phase PMSM windings is shown in Fig 1. In this figure, ABC is the first set of windings, and XYZ represents its second set. The windings of both sets are distributed at a 30° electrical angle from each other, resulting in what is known as "asymmetric six-phase motor". These, the two sets of windings are star-connected, and the neutral points are independent one from the other. Due to the particular distribution of the space structure of both sets of windings, the 5^th^ and 7^th^ order space harmonics are eliminated in the motor, whereas the 6^th^ order torque fluctuation is also canceled, so that the torque fluctuation reaches the minimum 12^th^ order. This method is widely used because it can reduce the torque fluctuations, so this paper selects the dual three-phase PMSM as the research object.

**Fig 2:**
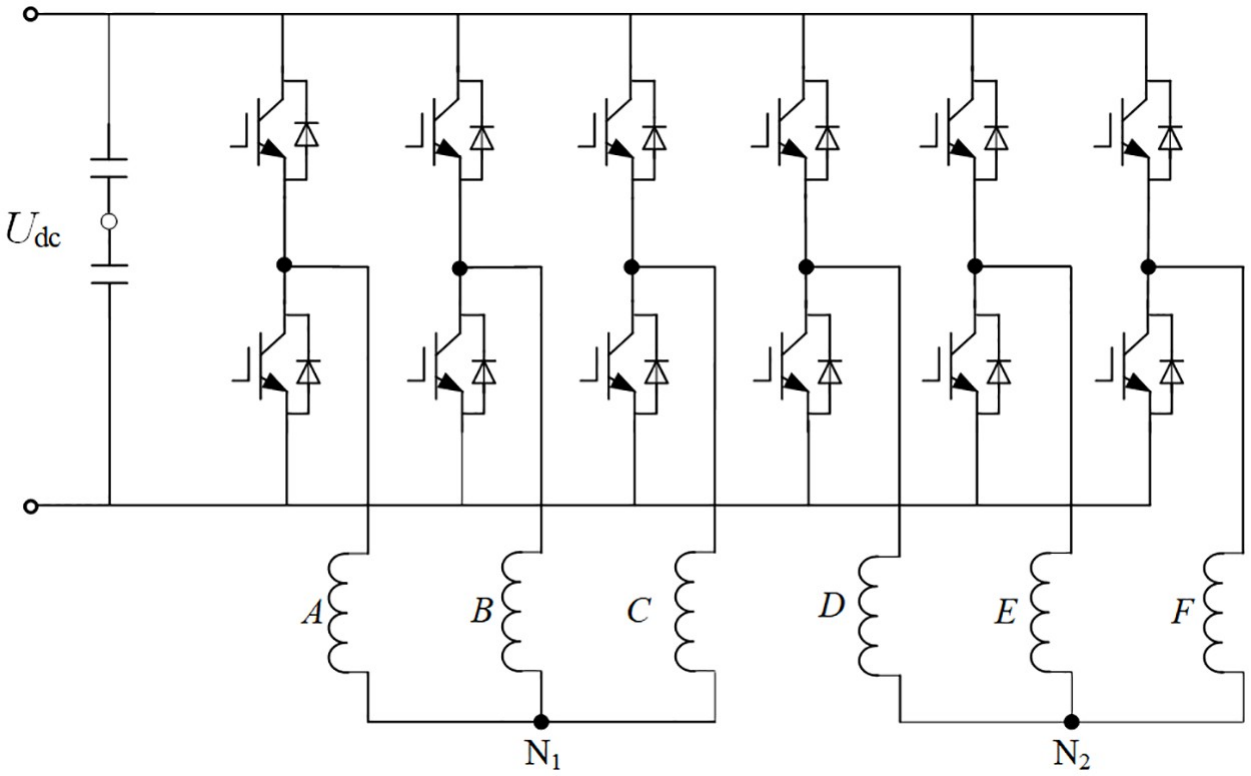
Asymmetric six-phase PMSM winding space structure diagram.

The power topology structure of the dual three-phase PMSM inverter is shown in Fig 2. The voltage-based power topology is adopted. The arm is the first set of windings, the neutral point is represented by *N1*, the last three bridge arms represent the second set of windings, and the neutral point is represented by *N2*.

The dual three-phase PMSM is a six-dimensional system. Compared to the traditional three-phase motor, its mathematical system model in the standard coordinate system has some defects, for instance, higher order of the system, stronger system coupling, nonlinearity and so on. Meanwhile, the magnetic flux is proportional to the stator current, and it is related to the rotor position angle θ; therefore, it is tricky to have good control. Moreover, the natural coordinate system can be changed by the Vector Space Decoupling (VSD) method, and the transformation matrix is as follows:

$$\begin{array}{l} C_{6s/2s}={\left[\begin{array}{cccccc} \frac{\alpha }{\left|\alpha \right|} & \frac{\beta }{\left|\beta \right|} & \frac{z_{1}}{\left|z_{1}\right|} & \frac{z_{2}}{\left|z_{2}\right|} & \frac{o_{1}}{\left|o_{1}\right|} & \frac{o_{2}}{\left|o_{2}\right|} \end{array}\right]}^{\text{T}}\\ \;=\frac{1}{3}\left[\begin{array}{cccccc} 1 & -\frac{1}{2} & -\frac{1}{2} & \frac{\sqrt{3}}{2} & -\frac{\sqrt{3}}{2} & 0\\ 0 & \frac{\sqrt{3}}{2} & -\frac{\sqrt{3}}{2} & \frac{1}{2} & \frac{1}{2} & -1\\ 1 & -\frac{1}{2} & -\frac{1}{2} & -\frac{\sqrt{3}}{2} & \frac{\sqrt{3}}{2} & 0\\ 0 & -\frac{\sqrt{3}}{2} & \frac{\sqrt{3}}{2} & \frac{1}{2} & \frac{1}{2} & -1\\ 1 & 1 & 1 & 0 & 0 & 0\\ 0 & 0 & 0 & 1 & 1 & 1 \end{array}\right] \end{array}$$ (1)

Using the method of VSD coordinate change, the variables of the dual three-phase PMSM can be transformed into three orthogonal planes, which are α-β, x-y and zero-order subspaces. In the transformation matrix of Eq (1), the first two lines are the α-β subspace, expressed as a fundamental space, the third and fourth lines are the x-y subspaces, and the last two lines represent the zero-order subspaces. The energy conversion of electromechanical is only related to the fundamental wave plane, but independent of the x-y subspace and the zero-order subspace.

When these both subspaces are multiplied by the identity matrix and the value has not changed, the rotation coordinate transformation is performed on the α-β space.

Therefore, the new transformation matrix is shown below:

$$C_{2\text{s}/2\text{r}}=\left[\begin{array}{cccccc} \text{cos}\;\theta & \text{sin}\;\theta & 0 & 0 & 0 & 0\\ -\text{sin}\;\theta & \text{cos}\;\theta & 0 & 0 & 0 & 0\\ 0 & 0 & 1 & 0 & 0 & 0\\ 0 & 0 & 0 & 1 & 0 & 0\\ 0 & 0 & 0 & 0 & 1 & 0\\ 0 & 0 & 0 & 0 & 0 & 1 \end{array}\right]$$ (2)

where θ is the rotor electrical degree. Through the derived matrix, the following motor parameter equations and motion equations can be obtained.

Magnetic flux equation:

$$\left[\begin{array}{l} \psi_{\text{d}}\\ \psi_{\text{q}} \end{array}\right]=\left[\begin{array}{cc} L_{\text{d}} & \text{0}\\ \text{0} & L_{\text{q}} \end{array}\right]\left[\begin{array}{l} i_{\text{d}}\\ i_{\text{q}} \end{array}\right]+\psi_{f}\left[\begin{array}{l} 1\\ 0 \end{array}\right]$$ (3)

Voltage equation:

$$\left[\begin{array}{l} u_{\text{d}}\\ u_{\text{q}} \end{array}\right]=\left[\begin{array}{cc} R & \text{0}\\ \text{0} & R \end{array}\right]\left[\begin{array}{l} i_{\text{d}}\\ i_{\text{q}} \end{array}\right]+\frac{\text{d}}{\text{d}t}\left[\begin{array}{l} \psi_{\text{d}}\\ \psi_{\text{q}} \end{array}\right]+\omega_{\text{e}}\left[\begin{array}{l} -\psi_{\text{q}}\\ \psi_{\text{d}} \end{array}\right]$$ (4)

Electromagnetic torque equation:

$$T_{\text{e}}=3p[\psi_{f}i_{q}+(L_{d}-L_{q})i_{d}i_{q}]$$ (5)

Equation of motion:

$$T_{\text{e}}-T_{\text{L}}-B\omega =J\frac{\text{d}\omega }{\text{d}t}$$ (6)

where *L*_*d*_ and *L*_*q*_ are the inductances; *i*_*d*_ and *i*_*q*_ are the currents; *ψ*_*d*_ and *ψ*_*q*_ are the stator magnetic fluxes; *u*_*d*_ and *u*_*q*_ are the stator voltages and dq is expressed as d-axis and q-axis. In addition, *T*_*e*_, *T*_*L*_, *ψ*_*f*_, *B*, *ω*, *ω*_*e*_, *J*, *R*, and *p* correspond to rotor permanent magnetic flux, electromagnetic torque, load torque, mechanical angular velocity, electrical angular velocity, damping coefficient, motor inertia moment, stator resistance and polar logarithms, respectively.

## 3 Position sensorless detection technology with speed feedback

Compared to the traditional back-EMF method to calculate the rotor position and speed, this paper applies a new observer with speed feedback to estimate the sine and cosine curves of the motor. This novel method, removes the influence of the flux linkage and speed in the back-EMF, while providing the speed feedback. It can also reduce the estimation error and get a higher calculation accuracy.

The flux linkage equation of the dual three-phase PMSM is expressed as follows:

$$\left\{\begin{array}{c} \psi_{\alpha }=L_{s}i_{\alpha }+\frac{\psi_{f}}{p}\text{sin}\;p\theta \\ \psi_{\beta }=L_{s}i_{\beta }+\frac{\psi_{f}}{p}\text{cos}\;p\theta \end{array}\right.$$ (7)

And according to the voltage equation there are:

$$\left\{\begin{array}{c} {\dot{\psi }}_{\alpha }=-Ri_{\alpha }+u_{\alpha }\\ {\dot{\psi }}_{\beta }=-Ri_{\beta }+u_{\beta } \end{array}\right.$$ (8)

Combined with Eqs (7) and (8), the estimated sine and cosine curves are obtained:

$$\left\{\begin{array}{l} \text{sin}\;p\hat{\theta }=\frac{p}{\psi_{f}}\left({\hat{\psi }}_{\alpha }-L_{s}i_{\alpha }\right)\\ \text{cos}\;p\hat{\theta }=\frac{p}{\psi_{f}}\left({\hat{\psi }}_{\beta }-L_{s}i_{\beta }\right) \end{array}\right.$$ (9)

To find the estimated sine-cosine curve, the feedback input parameter *f* is introduced due to the angle estimation error as indicated below:

$$\begin{array}{l} f=\frac{\psi_{f}{}^{2}}{p^{2}}\left({\text{sin}}^{2}\;p\theta +{\text{cos}}^{2}\;p\theta -{\text{sin}}^{2}\;p\hat{\theta }-{\text{cos}}^{2}\;p\hat{\theta }\right)\\ \;=\frac{\psi_{f}{}^{2}}{p^{2}}\left(1-{\text{sin}}^{2}\;p\hat{\theta }-{\text{cos}}^{2}\;p\hat{\theta }\right) \end{array}$$ (10)

In the formula: *sinpθ* and *cospθ* are the actual angles, the sum of squares is 1, *p* is the number of motor pole pairs, $\text{sin}p\hat{\theta },\text{cos}p\hat{\theta }$ is the estimated sine and cosine curve, and *f* is the angle error parameter generated by the actual and the estimated angles.

**Fig 3:**
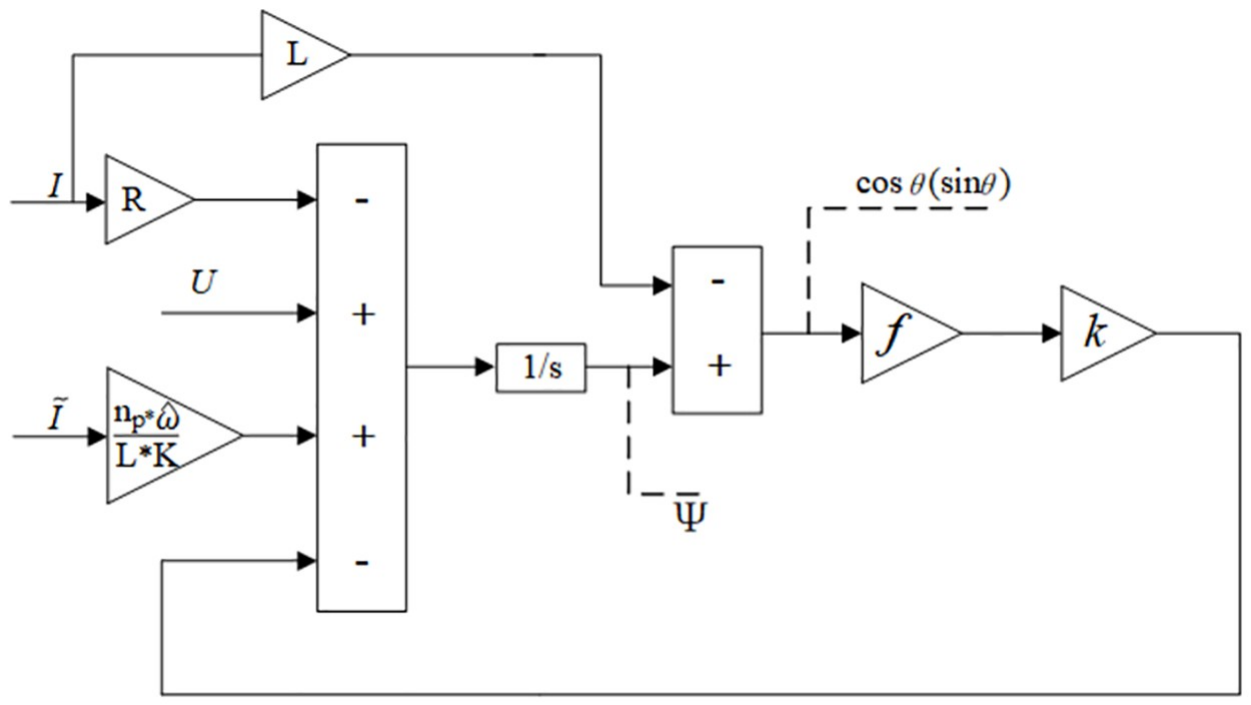
Functional block diagram of magnetic flux observer.

Building a flux observer is achieved based on the angle estimation error parameter and the estimated velocity feedback: based on angle estimation error parameters and estimation speed feedback, a magnetic flux observer is generated as follows, and its functional block diagram is shown in Fig 3.


$$\left\{\begin{array}{c} {\dot{\hat{\psi }}}_{\alpha }=-Ri_{\alpha }+u_{\alpha }+k_{\theta }\left({\hat{\psi }}_{\alpha }-Li_{\alpha }\right)f-\frac{p\hat{\omega }}{Lk}{\tilde{i}}_{\beta }\\ {\dot{\hat{\psi }}}_{\beta }=-Ri_{\beta }+u_{\beta }+k_{\theta }\left({\hat{\psi }}_{\beta }-Li_{\beta }\right)f-\frac{p\hat{\omega }}{Lk}{\tilde{i}}_{\alpha } \end{array}\right.$$ (11)

In Eq (11): *k*, *k*_*θ*_ are positive gain parameters, $\hat{\omega }$ is the estimated speed feedback value, and ${\tilde{i}}_{\alpha },{\tilde{i}}_{\beta }$ estimated the current errors that can be derived as follows.


$$\left\{\begin{array}{l} \frac{\text{d}i_{\alpha }}{\text{d}t}=-\frac{R}{L_{s}}i_{\alpha }+\frac{1}{L_{s}}u_{\alpha }+\frac{\psi_{f}}{L_{s}}\omega \;\text{sin}\;p\theta \\ \frac{\text{d}i_{\beta }}{\text{d}t}=-\frac{R}{L_{s}}i_{\beta }+\frac{1}{L_{s}}u_{\beta }-\frac{\psi_{f}}{L_{s}}\omega \;\text{cos}\;p\theta \end{array}\right.$$ (12)

The current observer is generated refereeing to Eq (12) and the FVT function is introduced to optimize the tracking system.

Let $u=[{\tilde{i}}_{\alpha },{\tilde{i}}_{\beta }]$ and $y=[{\hat{e}}_{\alpha },{\hat{e}}_{\beta }]$, then the FVT function can be expressed as:

$$F()=\frac{Y(s)}{U(s\text{)}}=\frac{K_{P}[s^{\text{2}}+\text{2}\omega_{c}s+{\left(2\pi f\right)}^{\text{2}}]\text{+}K_{\text{r}}s}{s^{\text{2}}+\text{2}\omega_{c}s+{\left(2\pi f\right)}^{\text{2}}}$$ (13)

Eq (13) reflects that the main parameters affecting the performance of the FVT function are *K*_*p*_, *K*_*r*_, *ω*_*c*_ and *f*. Where *K*_*p*_, *K*_*r*_ represent the scale factor and the peak gain at the resonant frequency, respectively, whereas *f* is the estimating frequency of the stator current, and *ω*_*c*_ affects the resonance gain and bandwidth. Adjusting each parameter will yield to different behaviors. Among them, regulating *K*_*r*_ can reduce the steady state error and adjusting *ω*_*c*_ can control the fluctuation due to the frequency changes. During the experiment, each parameter should be adjusted in real-time.

**Fig 4:**
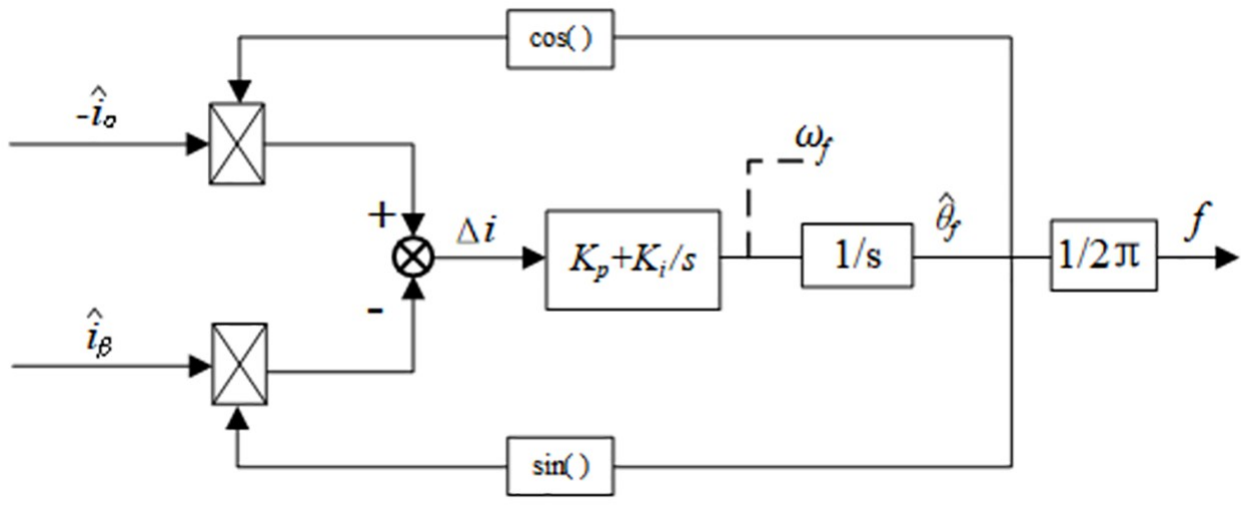
Block diagram of stator current frequency detection.

To track the changing frequency, the stator current frequency detection method based on the phase-locked loop displayed in Fig 4 is used to detect the stator current frequency.

According to the estimated value of the stator currents ${\hat{i}}_{\alpha },{\hat{i}}_{\beta }$, the current variation *Δi* is obtained as follows:

$$\Delta i=-{\hat{i}}_{\alpha }\;\text{cos}\;{\hat{\theta }}_{f}-{\hat{i}}_{\beta }\;\text{sin}\;{\hat{\theta }}_{f}$$ (14)

The current variation is adjusted using a Proportional-Integrator (PI) controller to obtain the angular frequency *ω*_*f*_:

$$\omega_{f}=(K_{p}+\frac{K_{i}}{s})\cdot \Delta i$$ (15)

As for the stator current frequency *f*, it is expressed as follows:

$$f=\frac{\omega_{f}}{\text{2}\pi }$$ (16)

In order to better cope with the frequency change and adapt to the simulation calculation process, it is necessary to convert the transfer function, expressed originally in the time domain into Z domain. The results are as follows:

$$\left\{\begin{array}{l} Y(\text{s})=[K_{p}{\text{s}}^{\text{2}}+(\text{2}K_{p}\omega_{c}+K_{r})s+K_{p}\omega_{e}{}^{\text{2}}]\cdot Z(\text{s})\\ U(\text{s})=({\text{s}}^{\text{2}}+\text{2}\omega_{c}s+\omega_{e}{}^{\text{2}})\cdot Z(\text{s}) \end{array}\right.$$ (17)

Which translates to:

$$\left\{\begin{array}{l} Y(z)=K_{p}\ddot{z}+(\text{2}K_{p}\omega_{c}+K_{r}\text{)}\dot{\text{z}}+K_{p}\omega_{e}{}^{2}\cdot z\\ U(\text{z})=\ddot{z}+2\omega_{c}\dot{z}+\omega_{e}{}^{2}\cdot z \end{array}\right.$$ (18)

Define state variables:

$$k=\left\{\begin{array}{l} k_{1}=z\\ k_{2}={\dot{k}}_{1}=\dot{z} \end{array}\right.$$ (19)

Deriving the derivative of Eq (19) yields the following state space equation:

$$\dot{k}=\left\{\begin{array}{l} {\dot{k}}_{1}=k_{2}\\ {\dot{k}}_{2}=-(2\omega_{c}k_{2}+\omega_{e}{}^{2}k_{1})+u \end{array}\right.$$ (20)

The FVT function obtained by Eq (20) is:

$$\left\{\begin{array}{l} \dot{k}=\left(\begin{array}{cc} 0 & 1\\ -\omega_{e}{}^{2} & -2\omega_{c} \end{array}\right)\cdot k+\left(\begin{array}{c} 0\\ 1 \end{array}\right)\cdot u\\ y=\left(\begin{array}{cc} 0 & K_{r} \end{array}\right)\cdot k+\left(\begin{array}{c} 0\\ K_{p} \end{array}\right)\cdot u \end{array}\right.$$ (21)

**Fig 5:**
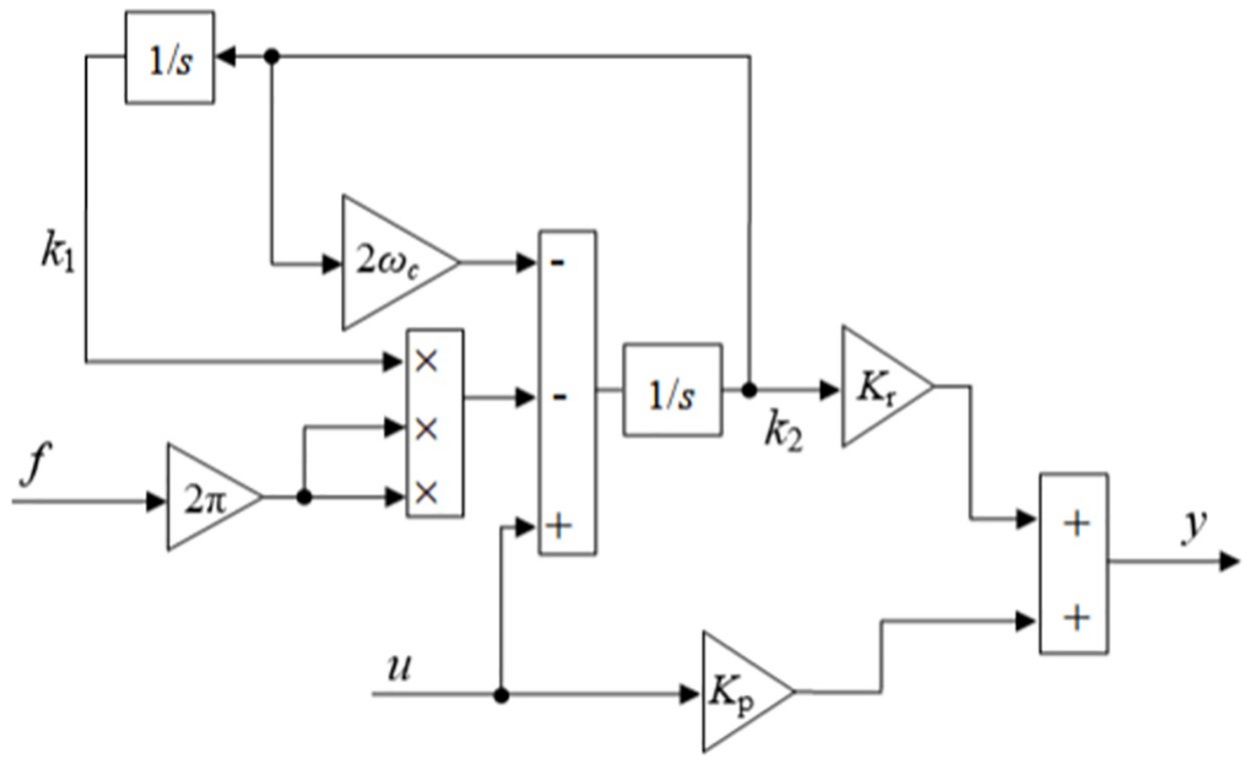
FVT function block diagram.

Through the above derivation equation, the functional block diagram of FVT is shown in Fig 5.

As the current error gain, the FVT function can track the current frequency and reduce the current error. Moreover, Eq (22) represents a current observer with frequency tracking constructed.


$$\left\{\begin{array}{l} {\dot{\hat{i}}}_{\alpha }=-\frac{R}{L_{s}}i_{\alpha }+\frac{1}{L_{s}}u_{\alpha }+\frac{\psi_{f}}{L_{s}}\hat{\omega }\;\text{sin}\;p\hat{\theta }+F({\tilde{i}}_{\alpha })\\ {\dot{\hat{i}}}_{\beta }=-\frac{R}{L_{s}}i_{\beta }+\frac{1}{L_{s}}u_{\beta }-\frac{\psi_{f}}{L_{s}}\hat{\omega }\;\text{cos}\;p\hat{\theta }+F({\tilde{i}}_{\beta }) \end{array}\right.$$ (22)

In this way, the estimated sine and cosine curves are obtained based on Eq (22), ${\tilde{i}}_{\alpha },{\tilde{i}}_{\beta }$ and $\hat{\omega }$ together with Eq (11), as shown in Eq (23).


$$\begin{array}{l} \left\{\begin{array}{l} {\dot{\hat{i}}}_{\alpha }=-\frac{R}{L_{s}}i_{\alpha }+\frac{1}{L_{s}}u_{\alpha }+\frac{\psi_{f}}{L_{s}}\hat{\omega }\;\text{sin}\;p\hat{\theta }+F({\tilde{i}}_{\beta })\\ {\dot{\hat{i}}}_{\beta }=-\frac{R}{L_{s}}i_{\beta }+\frac{1}{L_{s}}u_{\beta }-\frac{\psi_{f}}{L_{s}}\hat{\omega }\;\text{cos}\;p\hat{\theta }+F({\tilde{i}}_{\beta }) \end{array}\right.\\ \left\{\begin{array}{c} {\dot{\hat{\psi }}}_{\alpha }=-Ri_{\alpha }+u_{\alpha }+k_{\theta }\left({\hat{\psi }}_{\alpha }-Li_{\alpha }\right)f-\frac{p\hat{\omega }}{Lk}{\tilde{i}}_{\beta }\\ {\dot{\hat{\psi }}}_{\beta }=-Ri_{\beta }+u_{\beta }+k_{\theta }\left({\hat{\psi }}_{\beta }-Li_{\beta }\right)f-\frac{p\hat{\omega }}{Lk}{\tilde{i}}_{\alpha } \end{array}\right. \end{array}$$ (23)

Eq (23) represents a new observer constructed with estimated speed feedback and combined with the application of the FVT function to optimize the tracking current error. Furthermore, it is used to track and observe the sine and cosine curves in the back EMF, and it then apply the sine and cosine curves combined with ESO to estimate the speed and position of the rotor.

## 4 Rotor position estimation method based on ESO

In active disturbance rejection control, the ESO treats the total disturbance as a signal, expands it into a new first-order state variable, and uses an extended state observer for observation. For a Single-Input Single-Output (SISO) fourth-order system, the commonly used structure of the extended state observer is given by the following equation:

$$\begin{array}{l} {\dot{z}}_{1}=z_{2}+\beta_{h1}(\theta -z_{1})\\ {\dot{z}}_{2}=z_{3}+\beta_{h2}(\theta -z_{1})+bu\\ {\dot{z}}_{3}=z_{4}+\beta_{h3}(\theta -z_{1})\\ {\dot{z}}_{4}=\beta_{h4}(\theta -z_{1}) \end{array}$$ (24)

In the dual three-phase PMSM speed control system, the mechanical motion equation is shown in Eq (25):

$${\dot{\omega }}_{\text{e}}=\frac{n_{\text{p}}}{J}\left(T_{\text{e}}-T_{\text{L}}-B\omega_{\text{e}}\right)$$ (25)

Based on the relationship between the velocity and the rotor position angle in the motor motion equation, as well as the relationship between the rotor angle, angular velocity, and angular acceleration, a fourth-order ESO state equation can be established where the rotor position angle is designed as a coefficient, this state equation is expressed as follows:

$$\left\{\begin{array}{l} {\dot{\hat{\theta }}}_{e}={\hat{\omega }}_{e}+\beta_{h1}{\tilde{\theta }}_{e}\\ {\dot{\hat{\omega }}}_{e}=\frac{n_{\text{p}}}{J}(T_{\text{e}}-T_{L})+\frac{n_{\text{p}}}{J}a+\beta_{h2}{\tilde{\theta }}_{e}\\ \dot{a}=\frac{J}{n_{\text{p}}}Q+\beta_{h3}{\tilde{\theta }}_{e}\\ \dot{Q}=\beta_{h4}{\tilde{\theta }}_{e} \end{array}\right.$$ (26)

Where ${\tilde{\theta }}_{e}=\theta_{e}-{\hat{\theta }}_{e}$ is the estimated difference in rotor angle, ${\hat{\omega }}_{e}$ is the estimated rotor angular velocity, a is the motor’s angular acceleration, Q is the total disturbance quantity of the system, *β*_*h*1_, *β*_*h*2_, *β*_*h*3_ and *β*_*h*4_ is the output correction factor of the observation error, and *β*_*hi*_ > 0.

**Fig 6:**
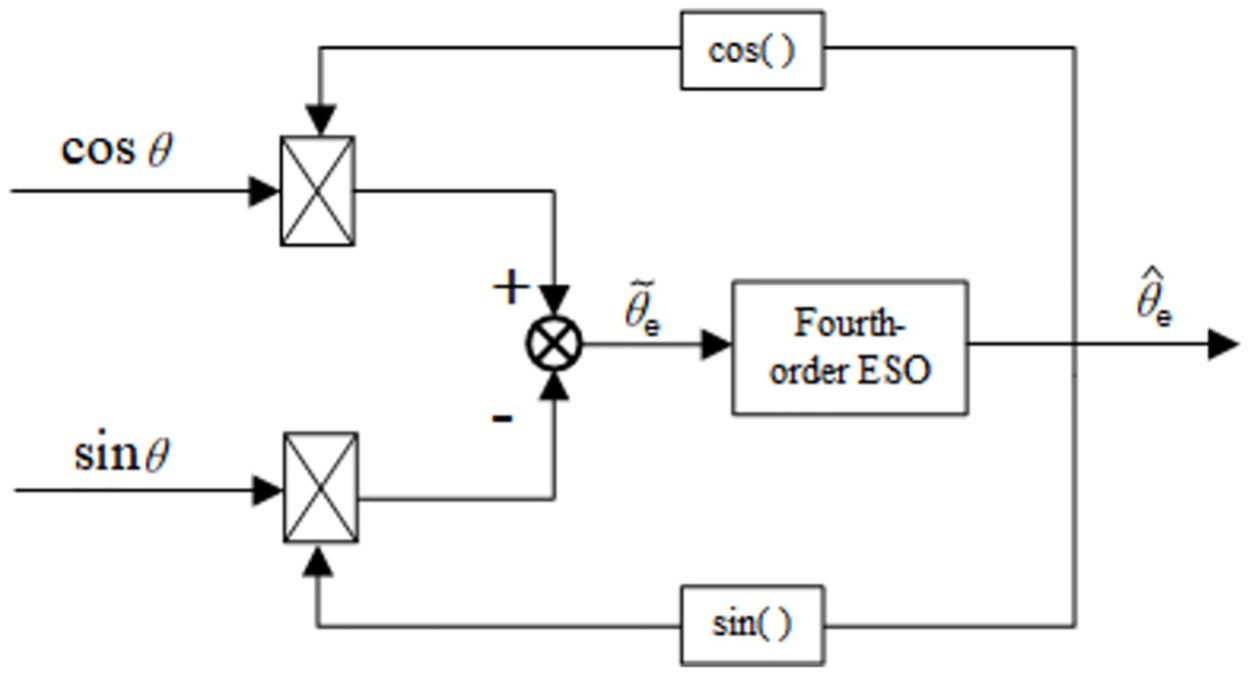
Block diagram of rotor position estimation based on ESO.

According to the relationship between the rotor position angle error and the fourth-order ESO, the structure shown in Fig 6 is used to estimate the rotor position angle.

According to the block diagram there following calculation can be derived:

$$\begin{array}{ll} {\tilde{\theta }}_{e} & =\text{sin}\;p\theta_{e}\;\text{cos}\;{\tilde{\theta }}_{e}-\text{cos}\;p{\tilde{\theta }}_{e}\;\text{sin}\;{\tilde{\theta }}_{e}\\ & =\text{sin}\;\theta_{e}\;\text{cos}\;{\tilde{\theta }}_{e}-\text{cos}\;{\tilde{\theta }}_{e}\;\text{sin}\;{\tilde{\theta }}_{e}\\ & =\text{sin(}\theta_{e}-{\tilde{\theta }}_{e})\approx (\theta_{e}-{\tilde{\theta }}_{e}) \end{array}$$ (27)

In the equation: when $\left|\theta_{e}-{\hat{\theta }}_{e}\right|<\pi /6$,$\text{sin}(\theta_{e}-{\hat{\theta }}_{e})\approx \theta_{e}-{\hat{\theta }}_{e}$.

Based on Eq (27), it is clear that, when the rotor position angle deviation is null (${\tilde{\theta }}_{e}=0$), the estimated rotor position angle is equal to the actual value $\theta_{e}={\hat{\theta }}_{e}$. Therefore, the real rotor position angle information can be obtained by observation.

**Fig 7:**
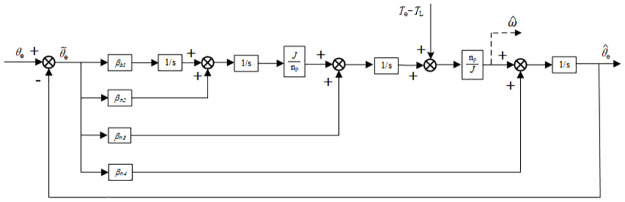
Structural flow chart of rotor position angle calculation based on ESO.

Moreover, the block diagram of the rotor position angle estimation based on ESO is shown in Fig 7.

According to this block diagram, the closed-loop transfer function of the entire system can be obtained as follows:

$$\begin{array}{ll} G_{ESO}(s) & =\frac{\beta_{h1}s^{3}+\beta_{h2}s^{2}+\beta_{h3}s+\beta_{h4}}{s^{4}+\beta_{h1}s^{3}+\beta_{h2}s^{2}+\beta_{h3}s+\beta_{h4}}\\ & =\frac{\beta_{h1}s^{3}+\beta_{h2}s^{2}+\beta_{h3}s+\beta_{h4}}{{\left(s+\omega_{n}\right)}^{3}} \end{array}$$ (28)

For linear ESO, assigning the poles of the observer to -*ω*_*n*_ points, leads to get -*ω*_*n*_ is a quadruple pole. The values of *β*_*hi*_ can be obtained as follows: $\beta_{h1}=4\omega_{n},\beta_{h2}=6\omega_{n}^{2}$, $\beta_{h3}=4\omega_{n}^{3},\beta_{h4}=\omega_{n}^{4}$ where *ω*_*n*_ is the bandwidth of the linear ESO.

**Fig 8:**
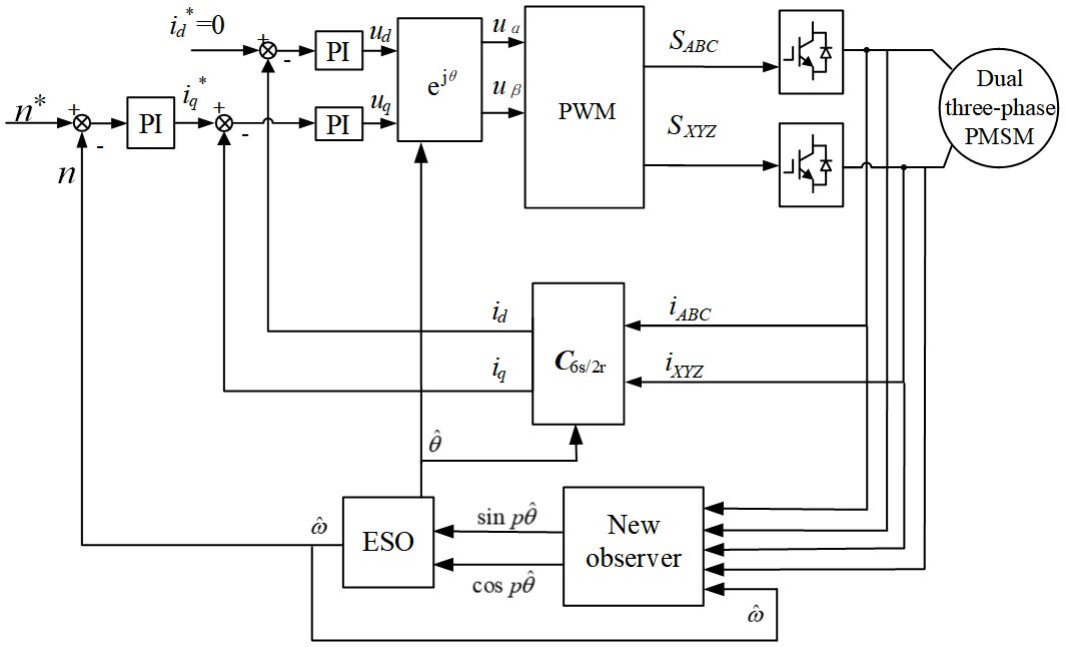
Block diagram of the position sensorless control system with speed feedback.

**Fig 9:**
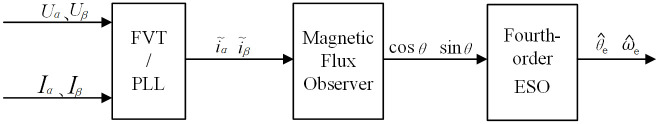
Structure diagram of the new observer.

As for Fig 8, it represents the structure diagram of the sensorless control system with speed feedback. The system adopts double a closed-loop control, where the inner loop is current PI control loop and the outer loop is the speed PI controlled speed loop. Moreover, speed sensor adopts a new observer with speed feedback, as shown in Fig 9. Monitoring the phase voltage and phase current signals of the motor through the new observer, as well as the sine and cosine signal with angle and speed information can be estimated by using the new observer. Furthermore, the rotational speed and rotor position are estimated by ESO, and the estimated rotational speed is feedback to the new observation used to improve the estimation accuracy.

## 5 Simulation analysis

The correctness of the sensorless control system with speed feedback is verified using MatLab© simulation. The dual three-phase PMSM simulation parameters used in this paper are shown in Table 1.

**Table 1:** Dual three-phase PMSM simulation parameters.

Parameter	Value
R(Ω)	0.1
Ld(mH)	0.82
Lq(mH)	0.82
Ψf(Wb)	0.072
J(kg∙m2)	0.015
B(N∙m∙s)	0
np	3

In the simulation model, the DC bus voltage is equal to 537 V, the PWM switching frequency is selected at 10kHz, the speed is given 1000r/min, the initial load torque is 20N∙m, and the load torque suddenly increases to 40N∙m within 2s. The simulation waveform is shown below.

**Fig 10:**
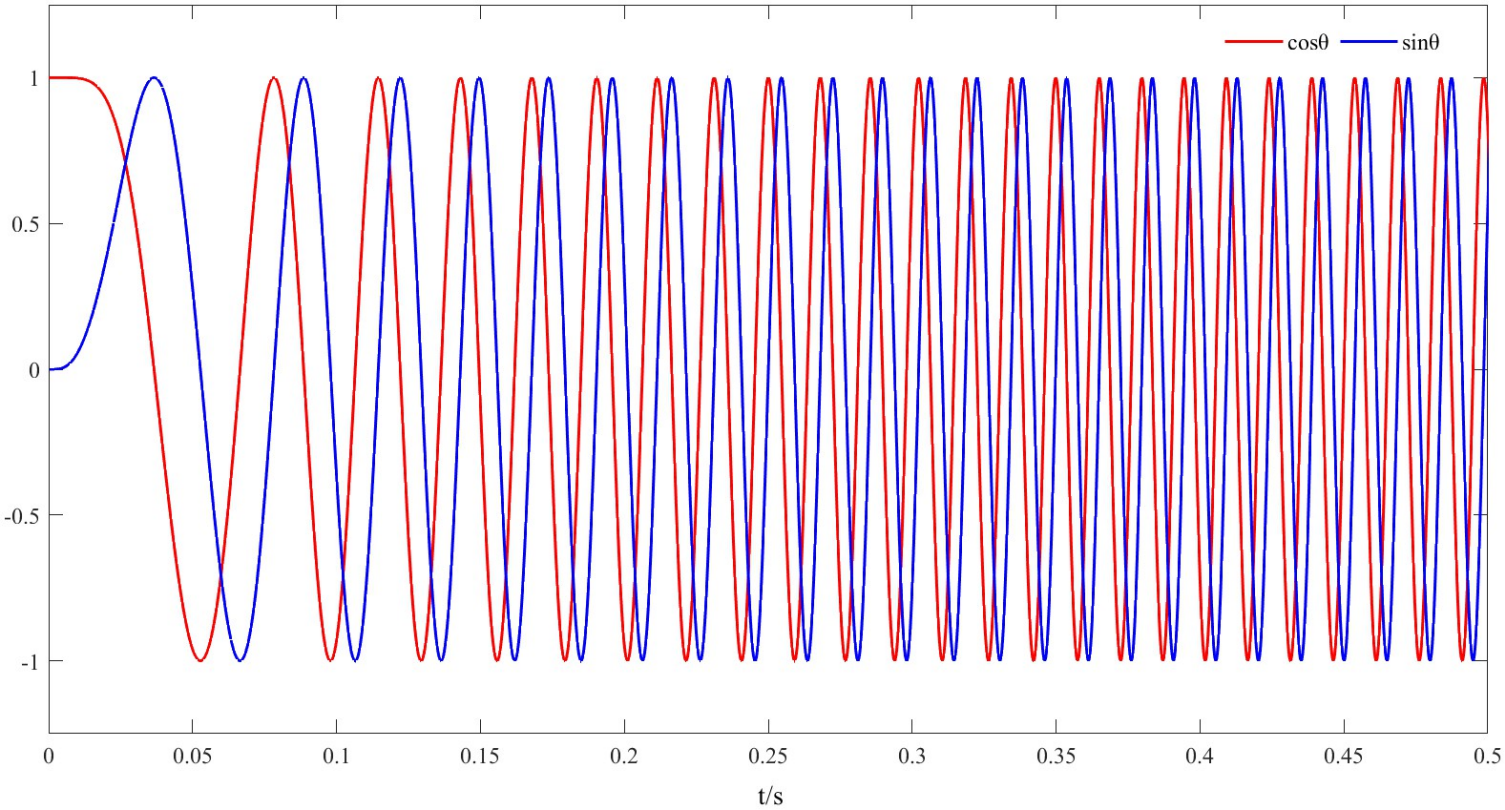
The sine and cosine curve of the new observer at 1000r/min, $\text{sin}\;p\hat{\theta },\;\text{cos}\;p\hat{\theta }$.

Fig 10 shows the sine and cosine curves observed by a new observer with speed feedback,$\text{sin}\;p\hat{\theta },\;\text{cos}\;p\hat{\theta }$. The amplitude of the curve in the figure varies between ±1. Compared to back EMF, the influence of the flux and speed are removed, at the same time, the speed feedback is introduced to make the estimation more accurate.

**Fig 11:**
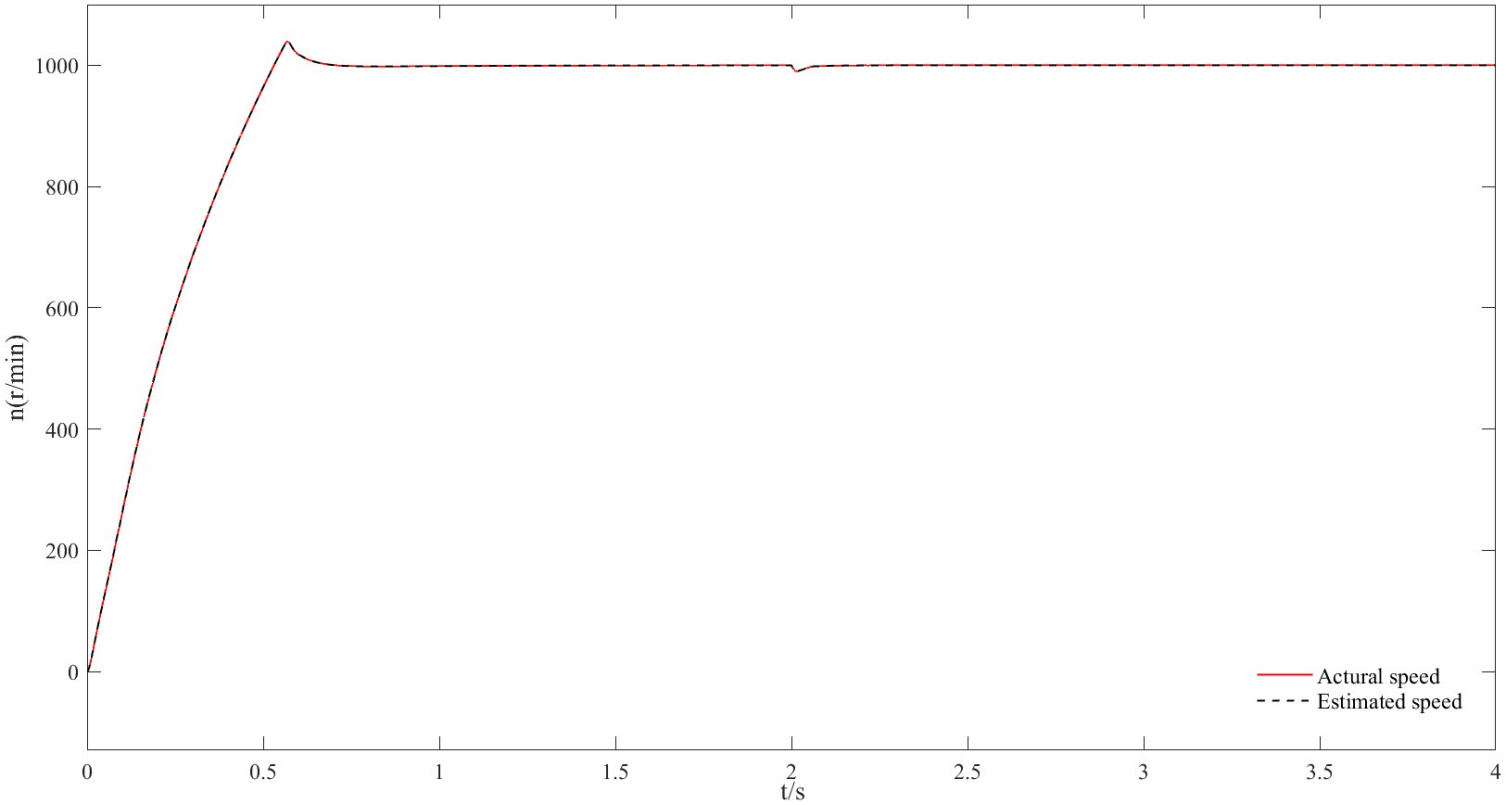
Speed waveform plot at 1000r/min.

**Fig 12:**
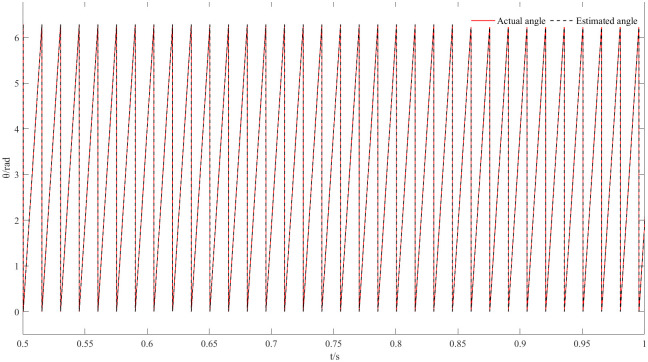
Angle waveform plot at 1000r/min.

**Fig 13:**
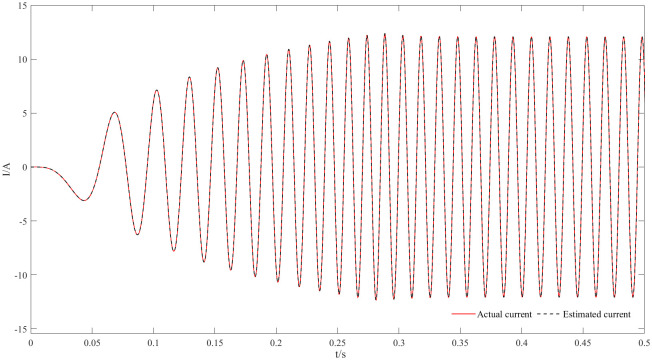
Current waveforms at 1000r/min.

**Fig 14:**
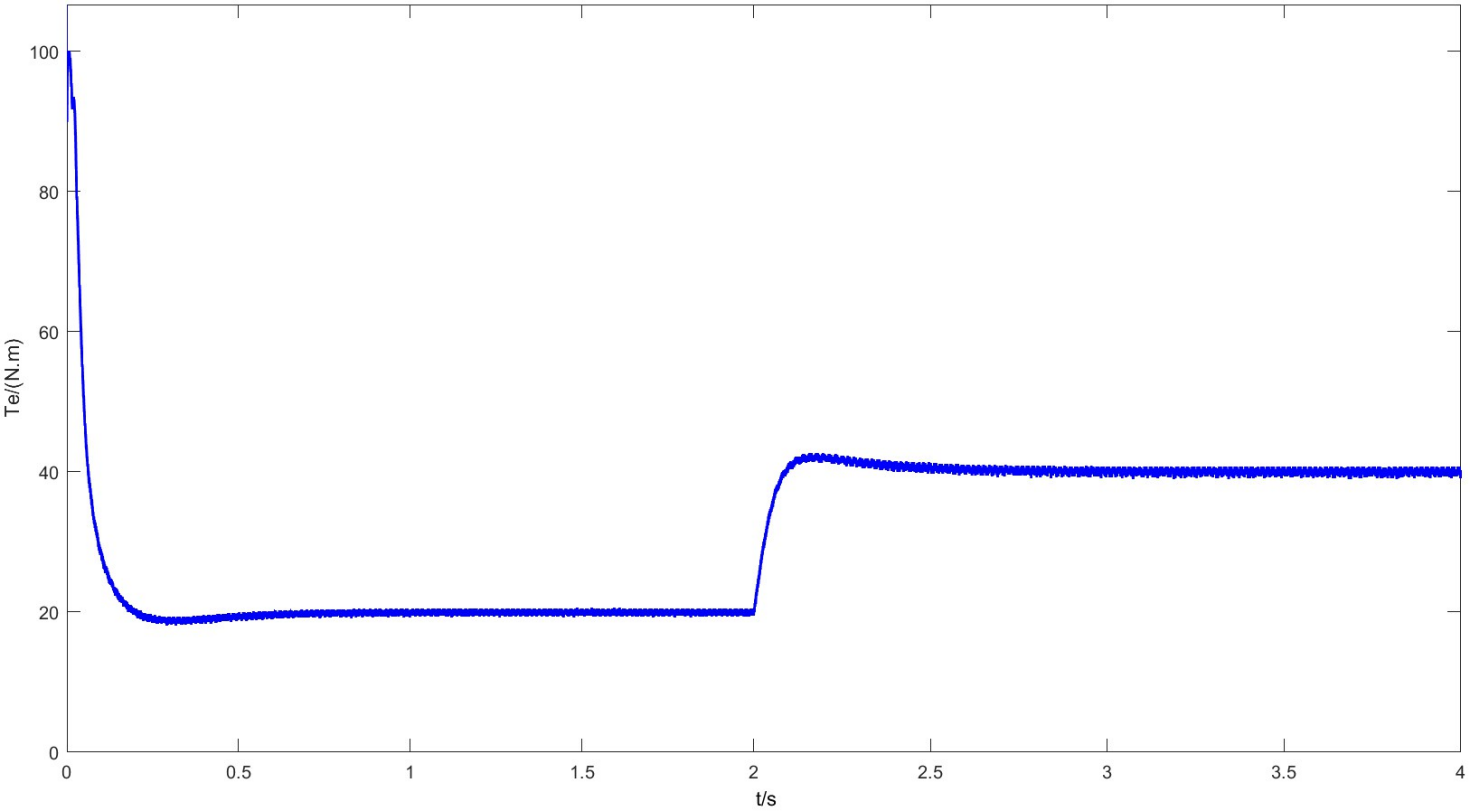
Electromagnetic torque waveforms at 1000r/min.

Figs 11 and 12 are speed and angle waveforms at 1000r/min. Based on Fig 11, the speed increases rapidly in the starting stage, when reaching the given speed, there is a certain overshoot, and the system’s output returns then to the steady state. The estimated speed can always follow the actual one, and the closed-loop effect is good when the steady state is reached. Referring to Fig 12, the estimated angle is highly consistent with the actual angle; therefore, it has always been able to follow the actual angle, and there is almost no phase delay. Moreover, Fig 13 represents the current waveforms at 1000r/min. At high speed, the current value, estimated by the new observer, has basically no deviation from the actual current value. Fig 14 shows the electromagnetic torque waveform at 1000r/min; in more detail, that the starting torque is large, then after reaching a constant speed, the torque returns to the given value. When the load of the motor abruptly reaches 40 N∙m at 2s, the output electromagnetic torque can quickly reach the steady state; following the load change, the dynamic response is good.

Next, the rotational speed dynamic simulation is performed where the given speed shifts from 800r/min to 1000r/min at medium speed and from 50r/min to 100r/min at low speed; therefore, and the simulated waveform is shown below.

**Fig 15:**
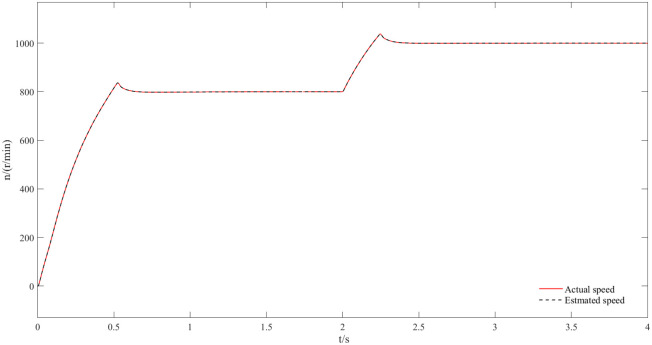
Dynamic speed waveforms at speeds from 800r/min to 1000r/min.

**Fig 16:**
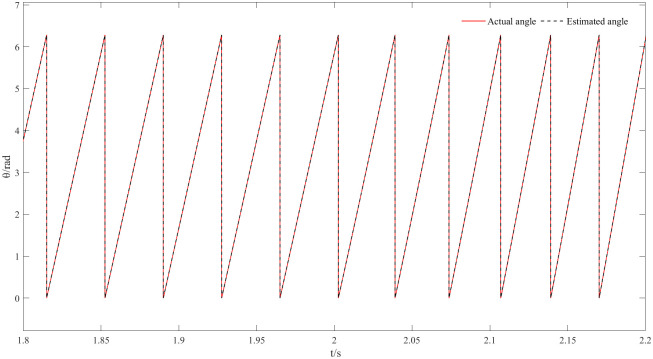
Dynamic angle waveforms at speeds from 800r/min to 1000r/min.

The speed and angle waveforms at medium speeds from 800r/min to 1000r/min are shown in Figs 15 and 16. Referring to Fig 15, the speed increases rapidly during the start-up phase where a certain overshoot is observed at the given speed, then gets back to the given speed and maintain the steady state. Simultaneously, the estimated speed can follow the actual speed; the closed loop works well at steady state. At 2s the given speed appears to change to 1000r/min, the motor responds quickly to this variation and the speed changes with the given value; moreover, the estimated value is consistent with the actual speed as it reflects the good dynamic effect of the new observer. Fig 16 compare the actual angle waveform to the estimated one, it can be also concluded that the two are always synchronized, the estimated angle always follows the actual angle and has almost no phase deviation.

**Fig 17:**
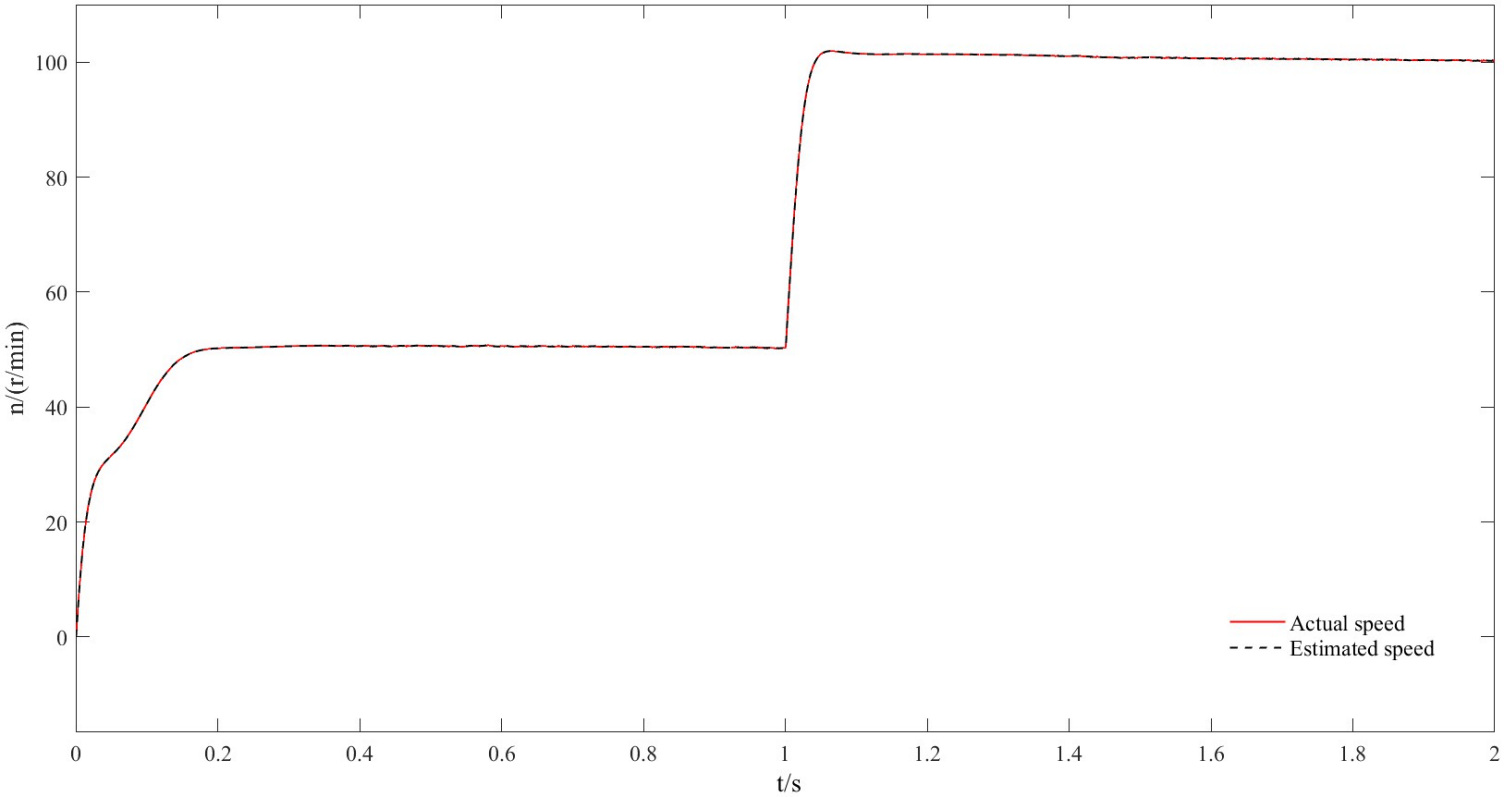
Dynamic speed waveforms at speeds from 50r/min to 100r/min.

**Fig 18:**
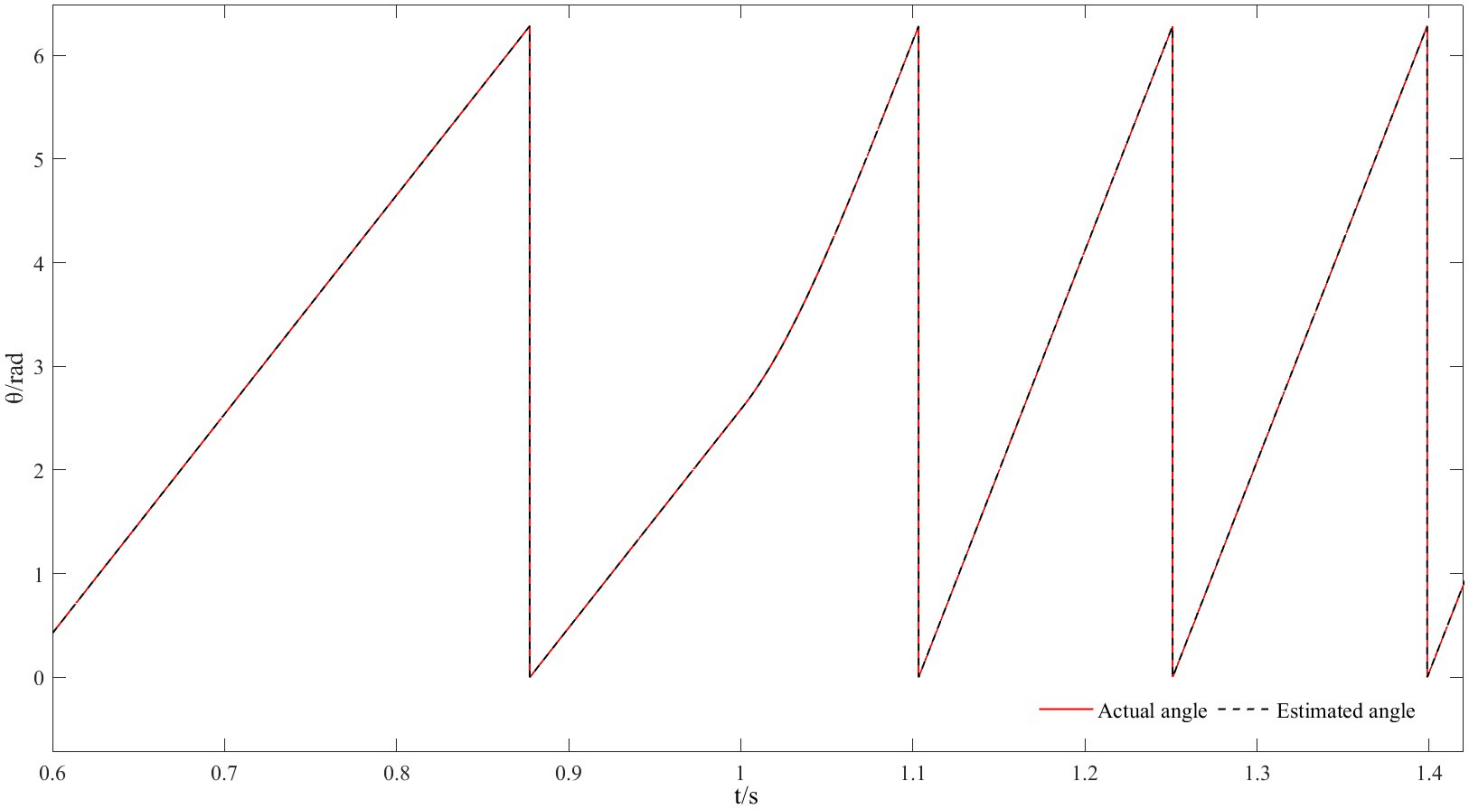
Dynamic angle waveforms at speeds from 50r/min to 100r/min.

Figs 17 and 18 represent the speed and angle dynamic change waveforms at low speeds from 50 r/min to 100 r/min. Fig 17 shows that the speed increases rapidly during the start-up phase, the steady-state is reached over some time and the overshoot is small. Furthermore, the estimated speed has basically no deviation from the actual speed and closed-loop effect is good at low steady state. At 1 s, the given speed appears to step change, and the motor can respond quickly and follow the given value; in the meantime, the estimated speed has always followed the actual speed, which reflects the good dynamic effect of the new observer at low speeds. By comparing the actual angle waveform with the estimated one, as visualized in Fig 18, it can be concluded that the both angles are still highly consistent at low speed where the estimated angle always follows the actual one with almost no phase delay.

The simulation analyses of the motor are carried out at low speed, The motor starts with load, the given speed is 20r/min and the load torque is 20N∙m, simulated waveforms are shown below.

**Fig 19:**
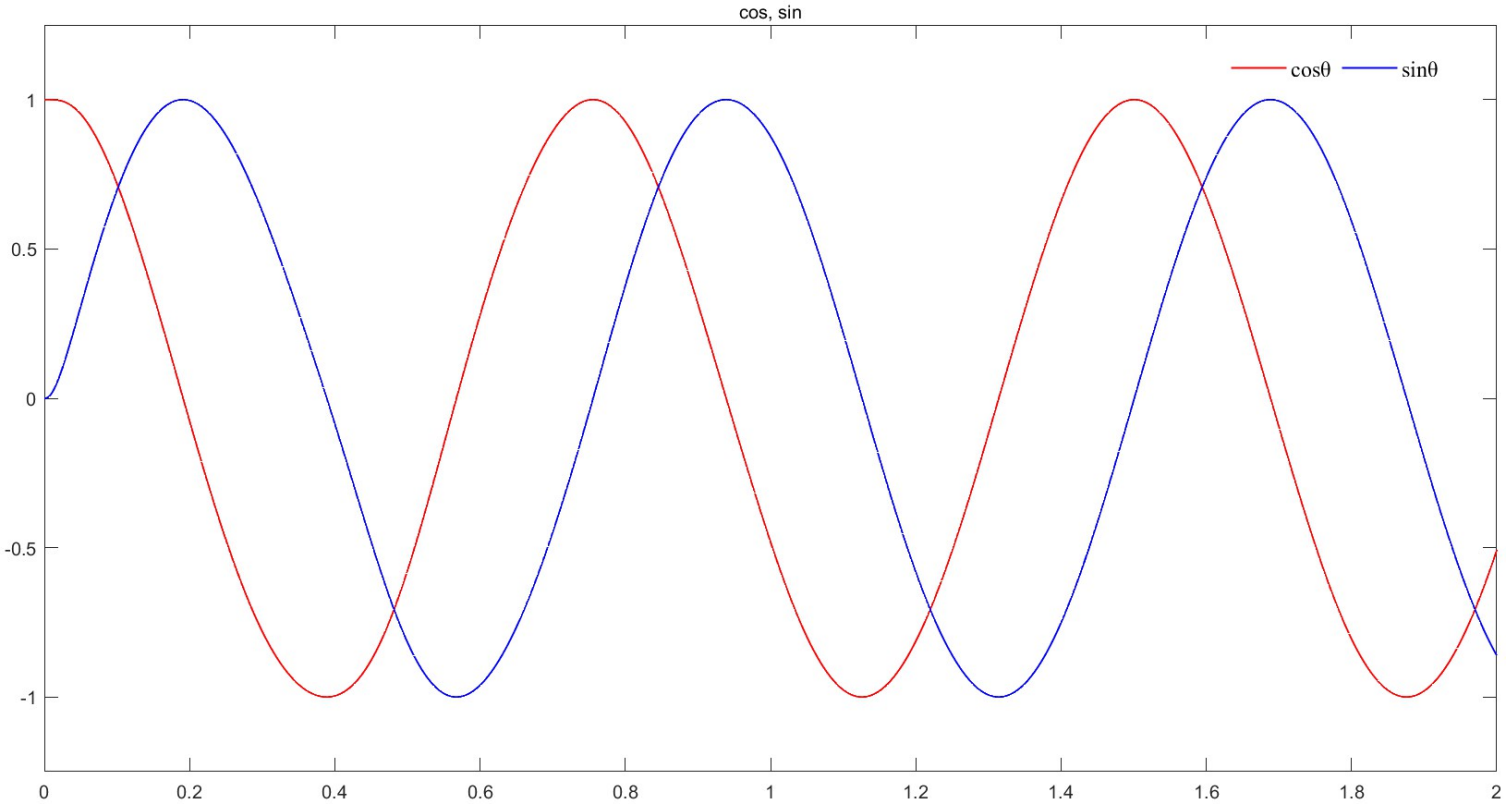
The sine and cosine curve of the new observer at 20r/min, $\text{sin}\;p\hat{\theta },\;\text{cos}\;p\hat{\theta }$.

Fig 19 represents the sine and cosine curve observed by the new observer with speed feedback at 20r/min,$\text{sin}\;p\hat{\theta },\;\text{cos}\;p\hat{\theta }$. The amplitude of the curve in the figure is ±1, the phase difference between $\text{sin}\;p\hat{\theta },\;\text{cos}\;p\hat{\theta }$ is 90°. Compared to the back EMF, the influence of the magnetic chain and speed at low values are removed, the speed feedback is introduced to make the estimation more accurate and sine degree is higher.

**Fig 20:**
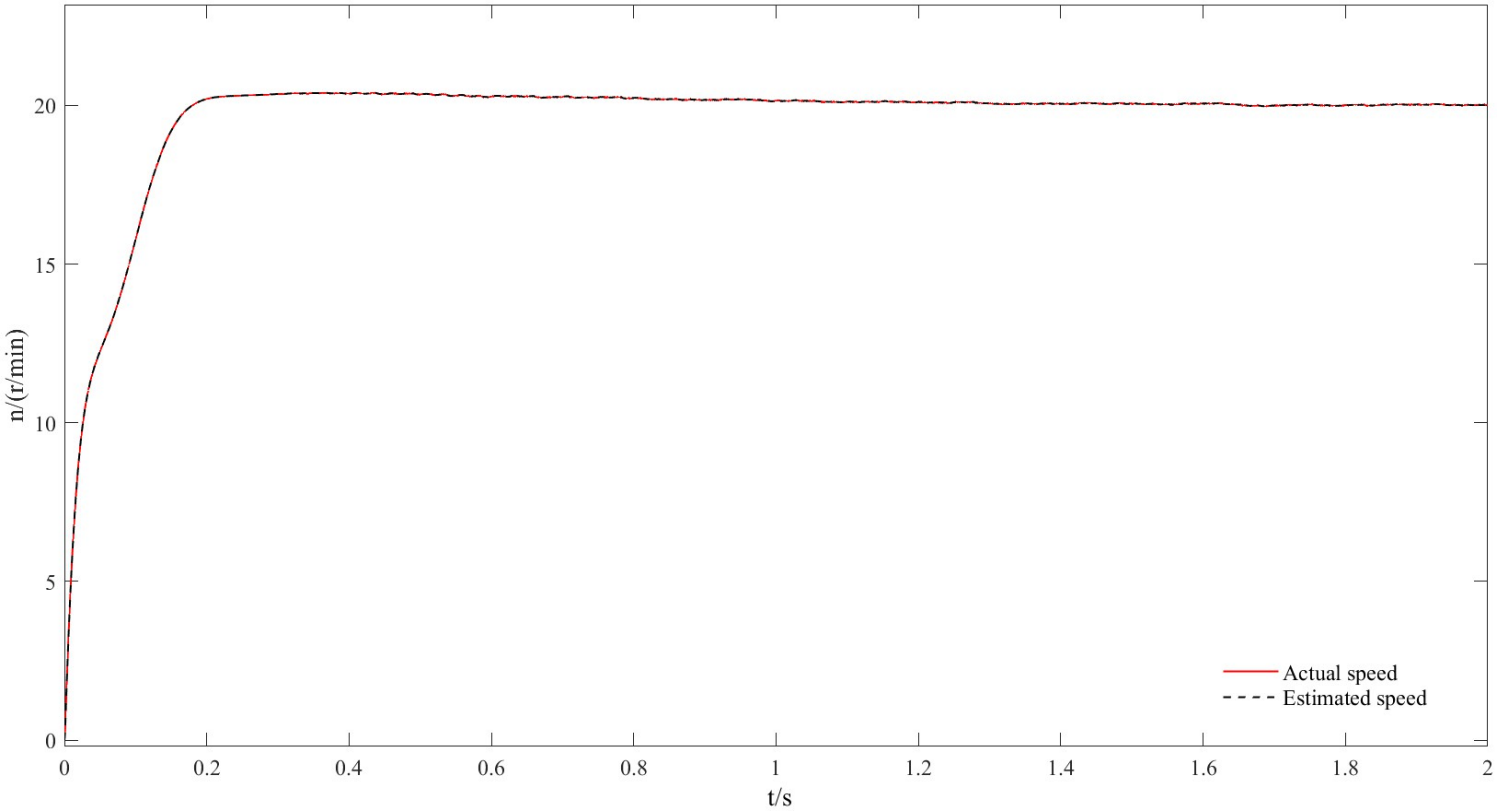
Speed waveform at 20r/min.

**Fig 21:**
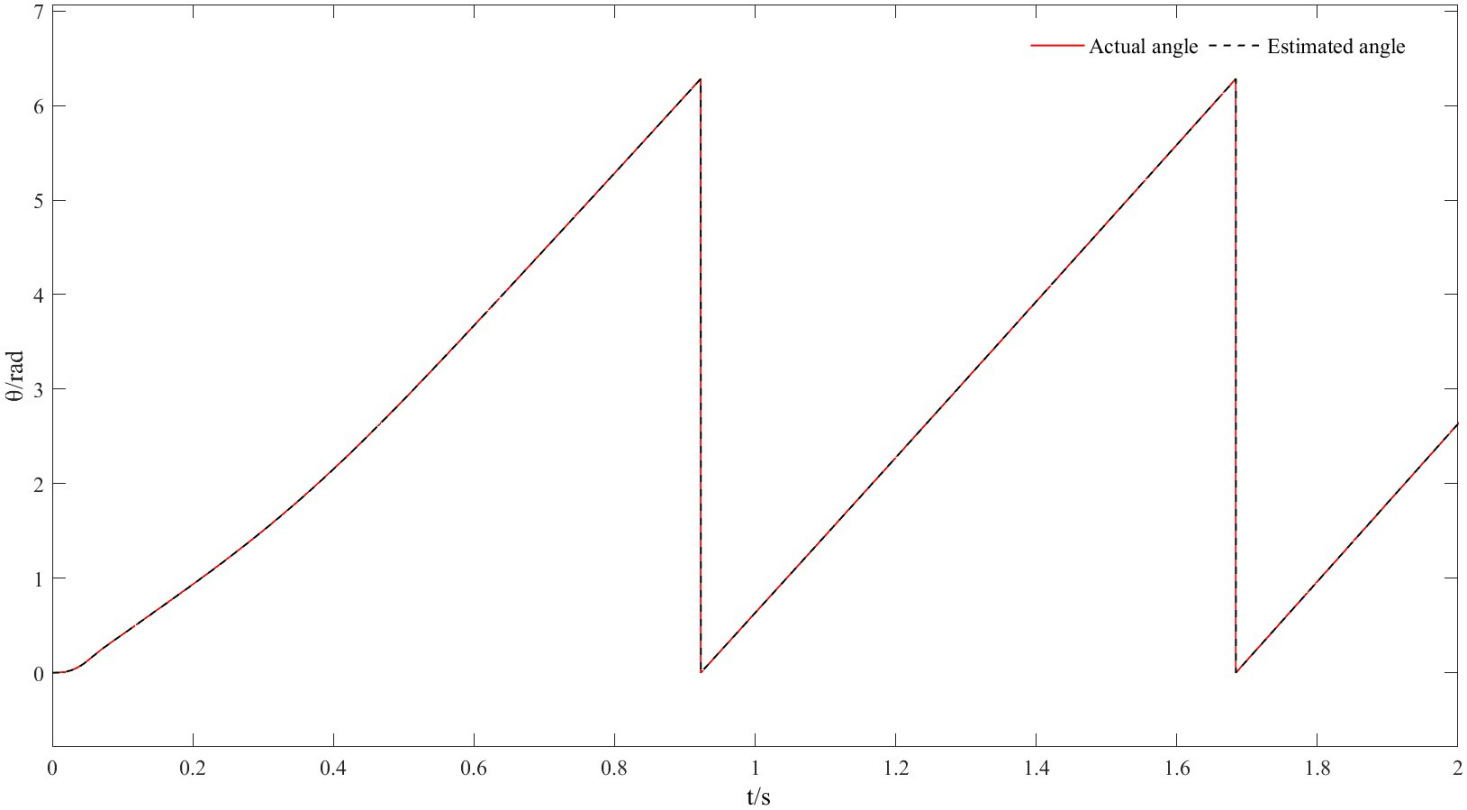
Angle waveform at 20r/min.

**Fig 22:**
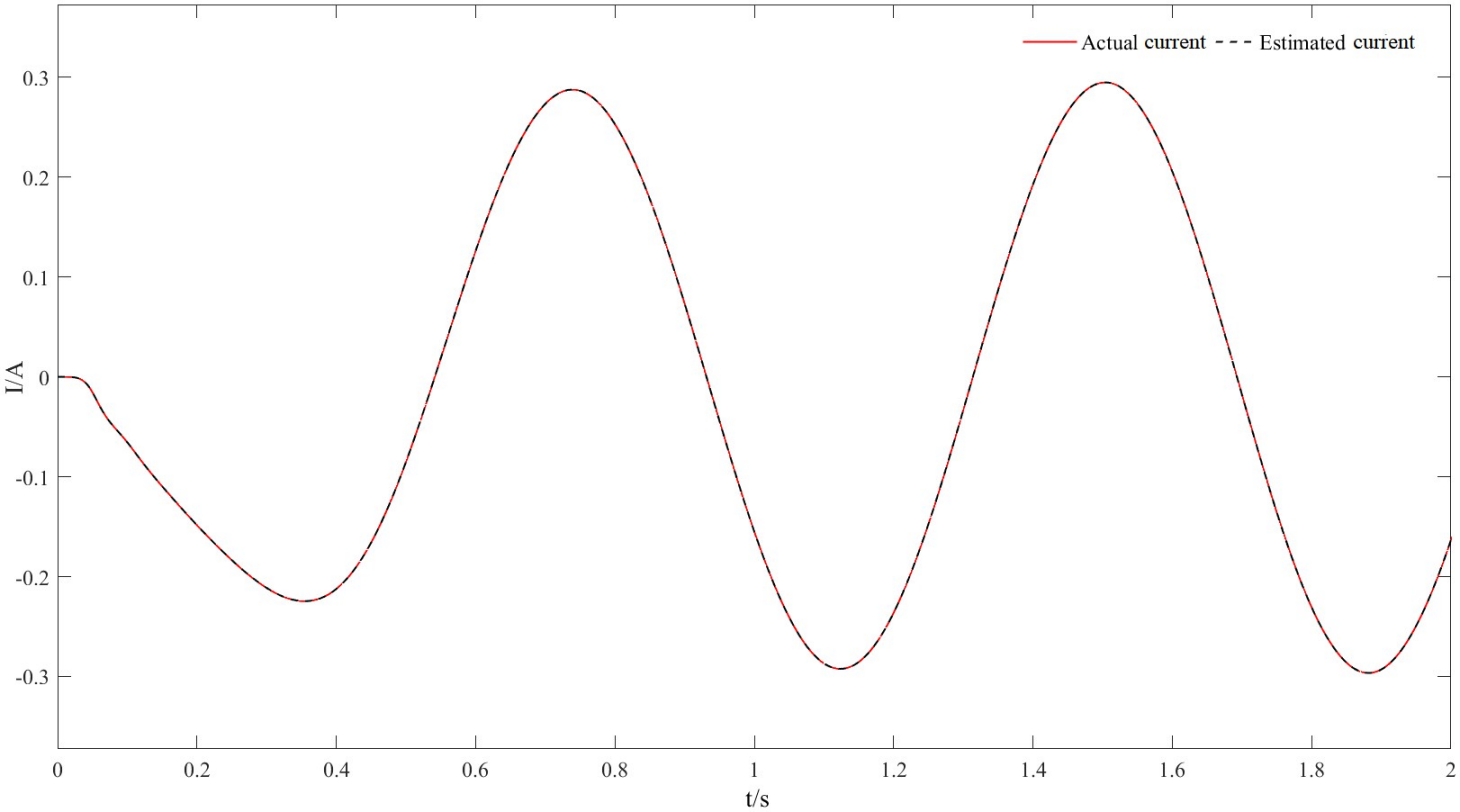
Current waveforms at 20r/min.

**Fig 23:**
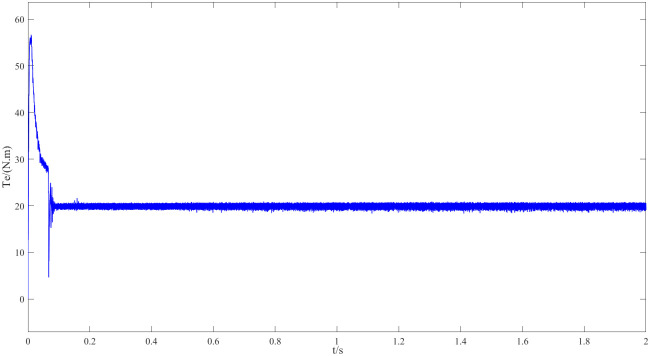
Electromagnetic torque waveform at 20r/min.

Figs 20 and 21 represent 20r/min rotational speed and angle waveforms at a low-speed steady state. In more detail Fig 20 shows that the speed increases smoothly and slowly during the start-up phase, and it reaches the steady state within some time with a small overshoot. Moreover, the estimated speed can follow the actual speed, basically error-free, and the closed-loop effect is good during the steady state. The angular waveform of Fig 21 can also verify this conclusion: the estimated position always follows the actual position, and the steady state effect is good at low speeds. Fig 22 represents the current waveforms at 20r/min. It can be seen that the new observer can still accurately estimate the current at low speed, overcoming the limitations of the traditional observer at low speed. Fig 23 represents the electromagnetic torque waveform at 20 r/min, the output electromagnetic torque can follow a given value and always remain stable, which also indicates a good carrying capacity at low-speed.

Through these low and medium speed simulations, the applicability and feasibility of the new speed feedback observer in the sensorless control system are proved.

## 6. Experimental verification

In the following, the sensorless detection technology described above is verified through experiments. The parameters of dual three-phase PMSM selected in this experiment are shown in Table 2.

**Table 2:** Dual three-phase PMSM parameters.

Parameter	Value
Rated Power(kw)	1.5
Rated Speed(r/min)	2000
Rated Voltage(V)	AC220
Rated Current(A)	4
Rated Frequency(Hz)	100
Stator resistance(Ω)	0.102Ω
D-axis inductance(mH)	0.82
Q-axis inductance(mH)	0.82
Number of pole-pairs	3
Flux(Wb)	0.072

**Fig 24:**
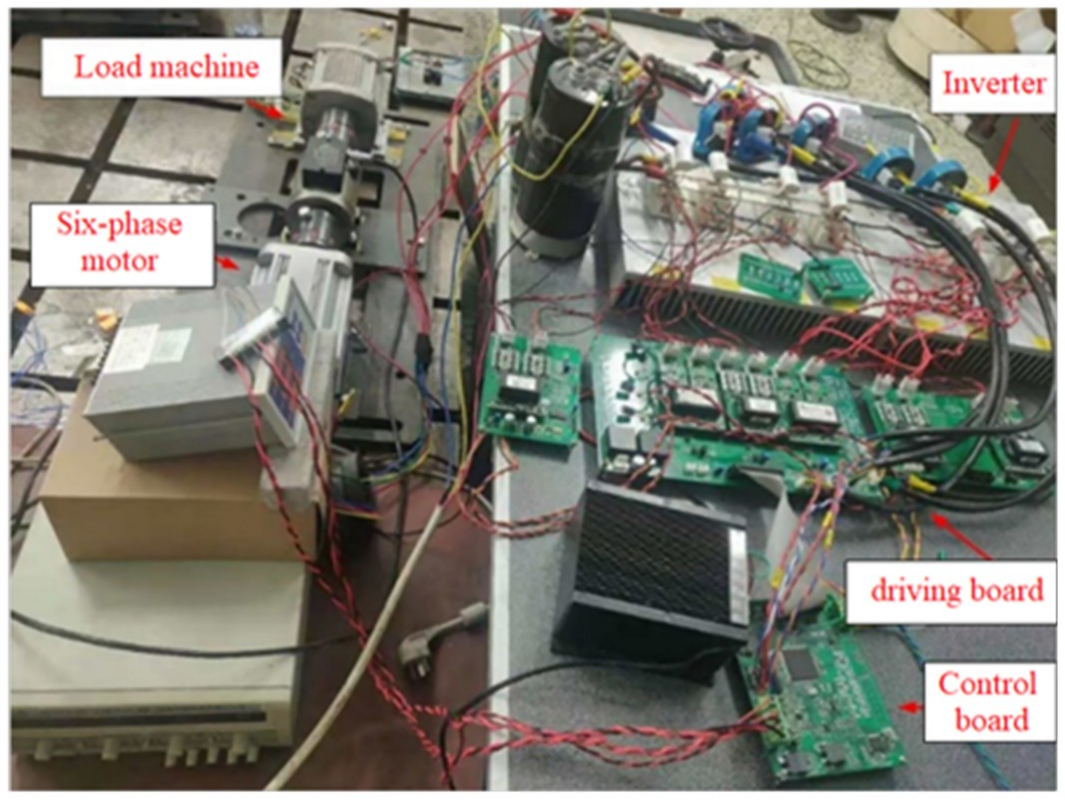
Experimental platform for dual three-phase PMSM control system.

The experimental platform for the dual-three-phase PMSM control system is shown in Fig 24.

In this experiment, the on-load operation of the dual three-phase PMSM is achieved by driving the synchronous generator. Firstly, the position, speed waveform, and sine and cosine waveforms of variable speed operation and the variable load operation in the dual three-phase motors are measured at high speed. Then, the same experiments are performed at low speeds to verify the reliability of the new observer in the sensorless control system.

**Fig 25:**
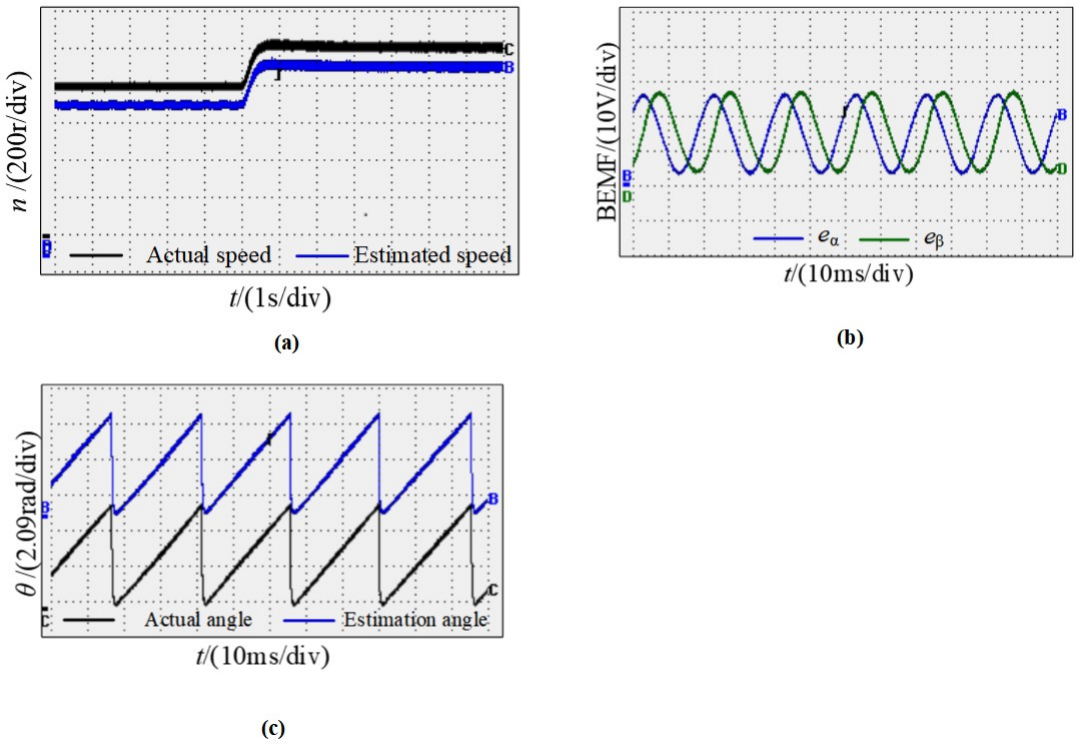
Dynamic waveform pattern of the motor from 800r/min to 1000r/min. (a) Speed waveforms; (b) Sine and cosine curves; (c) Angle waveforms.

Moreover, Fig 25(a) shows the speed waveform of the motor with starting at 800 r/min and then increasing to 1000 r/min, estimating the speed accurately follows the actual speed, and the estimated speed can track the change synchronously when the speed changes. Fig 25(b) shows the sine and cosine curve waveform measured by the new observe; it can be found that the phase difference of sine and cosine curves observed during motor operation is 90°, the waveform is smooth, and the estimation accuracy is high. Fig 25(c) shows the waveform between the actual rotor position angle and the observed value when the speed changes dynamically; the output shows that the position information of the observation is identical to the actual value, and there is basically no phase difference.

**Fig 26:**
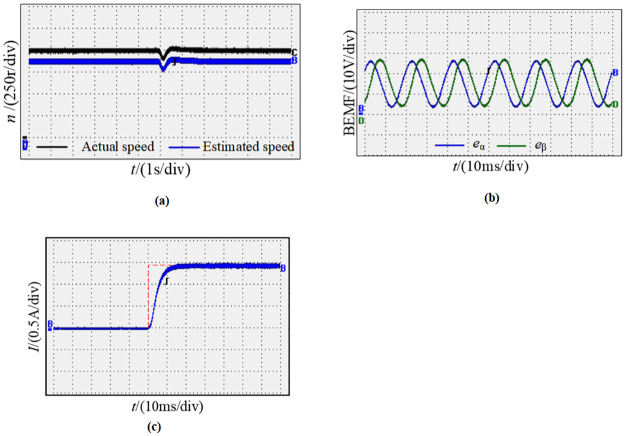
Waveforms of the motor at load change. (a) Speed waveforms; (b) Sine and cosine curves; (c) q-axis current waveform.

Fig 26 represents the waveform of the motor at 1000 r/min when the load changes. Fig 26(a) shows the motor speed waveform when the load changes rapidly from 20N.m to 40N.m. It can be seen that at the moment when the load changes, the estimated speed decreases synchronously with the actual speed and quickly returns to the steady state; thus, the new observer still has high accuracy in the case of variable torque. Fig 26(b) shows the sine and cosine curve waveform observed by the new observer under variable load conditions. It can be seen that the observed sine and cosine curves are basically not affected by the load change. Fig 26(c) presents the motor q-axis current waveform, the q-axis current is stable in steady state, and it is proportional to the output torque.

**Fig 27:**
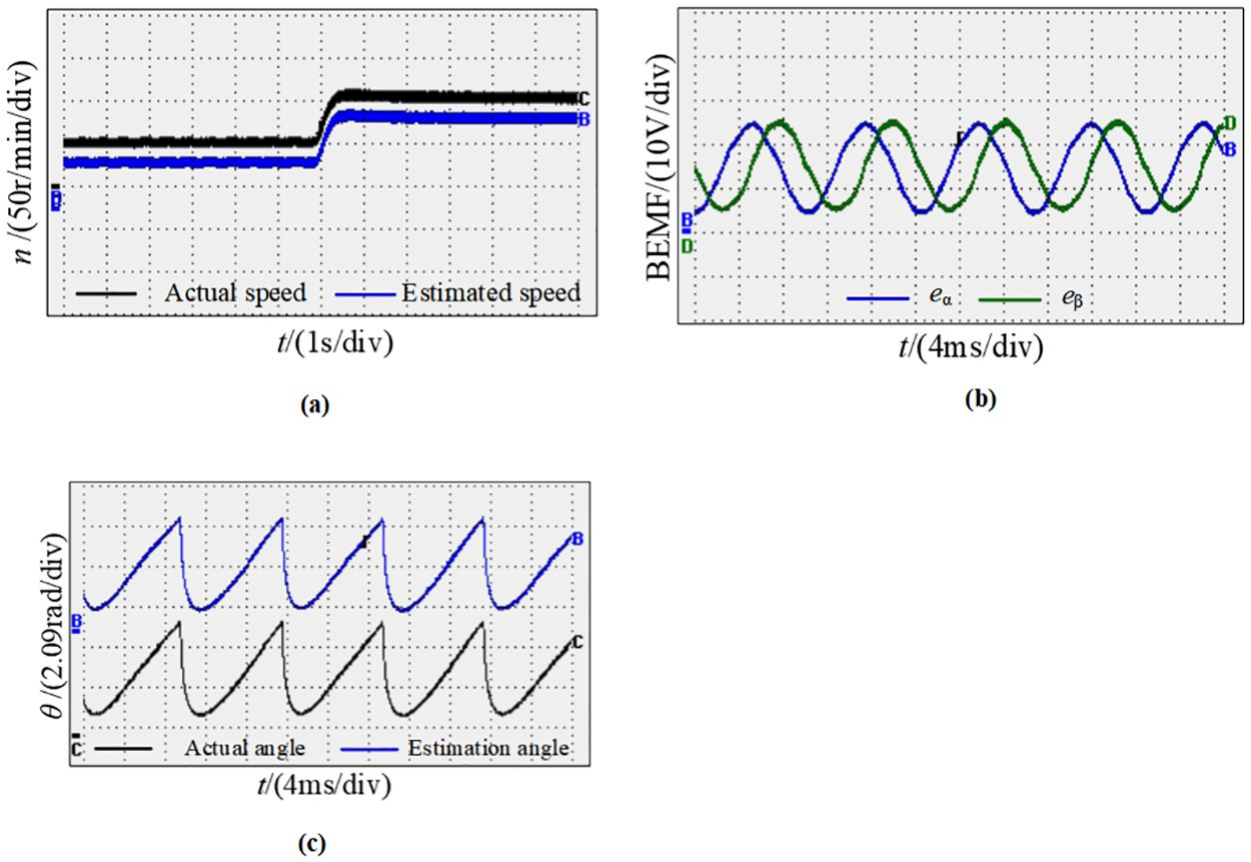
Dynamic waveforms pattern of the motor from 50r/min to 100r/min. (a) Speed waveforms; (b) Sine and cosine curves; (c) Angle waveforms.

Fig 27 represents the waveform diagram of the motor running at low speed, the initial speed is 50r/min, and then increased to 100r/min. Fig 27(a) shows the observed speed waveforms, it shows that in the case of low speed, the new observation system can still precisely calculate the actual speed, and the estimated speed can synchronously track the changes in the actual speed. Fig 27(b) is the sine and cosine curves observed by the new observer at low speeds, it can be seen that the sine and cosine curve waveforms are smooth at low speed, the accuracy is high, and there is no error caused by the low speed. Fig 27(c) shows the curve between the actual rotor position angle and the actual value when the motor is in low-speed dynamic operation. It can be concluded from the deviation that the estimated angle at low speed can always follow the actual angle, and there is basically no phase difference. From the above experiments, these can be concluded that experimental results are consistent with theoretical analysis and the simulation. Moreover, these experimental results verify the correctness and effectiveness of the new sensorless detection technology.

## 7. Conclusions

This paper proposed a sensorless detection technology based on speed feedback frequency conversion tracking. The sine and cosine signals with velocity and angle information are obtained by using the flux linkage observer, and the estimated angle error parameters are introduced to improve the accuracy by estimating the speed feedback. Moreover, the current frequency is tracked by the stator current FVT to reduce the current error. At the same time, to make the observer estimation more accurate and improve the ability of the observer to resist to disturbance, considering the rotor position angle error as the amount of deviation and through the mechanical motion equation of the motor, a fourth-order ESO was constructed to calculate the rotor position and speed. Through simulation and experiment, it is concluded that the proposed scheme has strong stability, small error, and it has a wide range of application, the accuracy is still high at low speed, solved the inapplicability of mathematical model method at low speed, which has certain engineering significance.
